# Is hysterectomy associated with kidney cancer risk? A meta-analysis of cohort studies

**DOI:** 10.3389/fonc.2023.1181112

**Published:** 2023-07-20

**Authors:** Ling Yu, Pengkui Yu, Yi Lu

**Affiliations:** Department of Urology, Shengzhou People’s Hospital, Shengzhou Branch of the First Affiliated Hospital of Zhejiang University, Shengzhou, Zhejiang, China

**Keywords:** hysterectomy, kidney cancer, cohort, meta-analysis, risk

## Abstract

**Introduction:**

Emerging evidence have suggested a potential relationship between hysterectomy and risk of kidney cancer with inconsistent results. We aimed to investigate the association of hysterectomy with kidney cancer risk based on a meta-analysis of all available cohort studies.

**Methods:**

A comprehensive literature search was performed in the PubMed and Embase database, covering all the papers published by September 2022. The pooled relative risks (RRs) and 95% confidence intervals (CIs) were estimated using a DerSimonian and Laird random effects model.

**Results:**

Overall, our meta-analysis included 10 cohorts from 9 studies with approximately 240 million participants. The pooled RR with its 95% CI showed a significantly positive association between hysterectomy and risk of kidney cancer (RR 1.30, 95% CI 1.19-1.41). No obvious heterogeneity was observed across the studies (*P* = 0.206 for heterogeneity; I^2^ = 25.9%).

**Conclusion:**

Findings from this meta-analysis of cohort studies indicated that hysterectomy was positively associated with subsequent kidney cancer risk. Further large prospective studies with long-term follow-up are warranted to verify these findings.

## Background

Kidney cancer develops from the renal parenchyma, which ranks seventh among the most frequently diagnosed cancers in men and ninth in women (1). According to 2018 GLOBOCAN data, approximately 403,000 people developed kidney cancer, constituting 2.2% of all cancer diagnoses, and an estimated 175,098 people died from kidney cancer, which accounted for 1.8% of all cancer deaths globally (2). The incidence of kidney cancer is two-fold higher in men compared with women (3). There was a slightly growing trend for both new kidney cancer incidence and mortality since 2012 (4). The average annual percentage increase is approximately 2% to 3% in most countries (5). These findings suggest that additional investigations are required for better prevention and treatment (6).

Established risk factors for kidney cancer include tobacco smoking, body size, history of hypertension and chronic kidney disease (7, 8). The difference in incidence by sex has motivated epidemiologic studies into the etiologic relevance of hormonal and reproductive factors. Emerging evidence have suggested a potential relationship between hysterectomy and risk of kidney cancer with conflicting results. Four relatively small prospective studies (9–12) performed before 2010 found no statistically significant relationship between hysterectomy and risk of kidney cancer. However, several large-scale cohort studies (13–15) with longer follow-up published since 2010 reported a positive relationship on this topic. Given the potential impact of hysterectomy on kidney cancer incidence, and inconsistent findings from previous studies, we, therefore, aimed to investigate the association of hysterectomy with kidney cancer risk based on a meta-analysis of all available cohort studies.

## Materials and methods

### Publication search

We conducted a comprehensive literature search in the PubMed and Embase database, covering all the papers published from their inception to September 2022. The search strategy was used as follows: (hysterectomy or reproductive factors) and (kidney or renal) and (cancer or carcinoma) and (cohort or prospective). We also examined the cited references from retrieved articles and reviews to identify additional relevant studies. This systematic review and meta-analysis was planned, performed, and reported according to the PRISMA guidelines of quality for reporting meta-analyses (16, 17).

### Study selection

Studies included in this meta-analysis met all of the following criteria: (a) one of the exposures of interest was hysterectomy history; (b) one of the outcomes of interest was kidney cancer incidence; (c) they had a cohort or prospective design; and (d) studies provided the rate ratios (RRs) or hazard ratios (HRs) with their corresponding 95% confidence intervals (CIs) or data to calculate them. Exclusion criteria: review or editorial paper; non-human studies; studies based on same population; cancer mortality as the outcome; and the exposure not including hysterectomy.

### Data extraction

Two authors (YL and LY) independently extracted the data using a predefined extraction form with disagreements resolved by consensus. The following characteristics were collected from each study: the first author’s name, year of publication, the country in which the study was performed, cohort name, cohort size, kidney cancer cases, average participants’ age, follow-up time, methods of ascertainment of hysterectomy and kidney cancer, risk estimates with their 95% CIs, and adjusted covariates in the data analysis. For each study, we extracted the most-fully adjusted risk estimate.

### Quality assessment

The same two authors (YL and LY) independently completed the quality assessment using the Newcastle-Ottawa Scale (NOS) (18), which was developed to assess the quality of observational studies with its design and content. NOS is an eight-item instrument with three broad perspectives and awards a maximum of nine points to each study. A higher score indicates better methodological quality. Any disagreements were resolved by consensus and discussion.

### Statistical methods

The pooled RRs and 95% CIs were estimated using a DerSimonian and Laird random effects model (19), which took account of both within- and between-study variability. The heterogeneity across studies was assessed by the *Q* statistic and the I^2^ score (20). The *Q* statistic was used to determine the presence of heterogeneity with a significance level set at *P* ≤ 0.10. The value of I^2^ was used to calculate the proportion of variation (I^2^ < 25% indicated low heterogeneity, I^2^ = 25-50% moderate heterogeneity, and I^2^ > 50% high heterogeneity). Galbraith plot analysis was further used to detect potential sources of heterogeneity. A number of stratified analyses were performed according to publishing year (before 2010 versus 2010 and thereafter), geographical region (Europe vs. United States), number of cases (> 500 vs. ≤ 500), number of participants (> 100,000 vs. ≤ 100,000), and duration of follow-up (> 15 years vs. ≤ 15 years). A sensitivity analysis was performed by repeating the meta-analysis after ruling out of each included study in turn. Potential publication bias was assessed by Begg’s test (21) and Egger’s test (22). All of the statistical analyses were performed using STATA 12.0 (StataCorp, College Station, TX). A 2-sided *P* value < 0.05 was considered significant unless stated otherwise.

## Results

### Literature search

**Figure 1:**
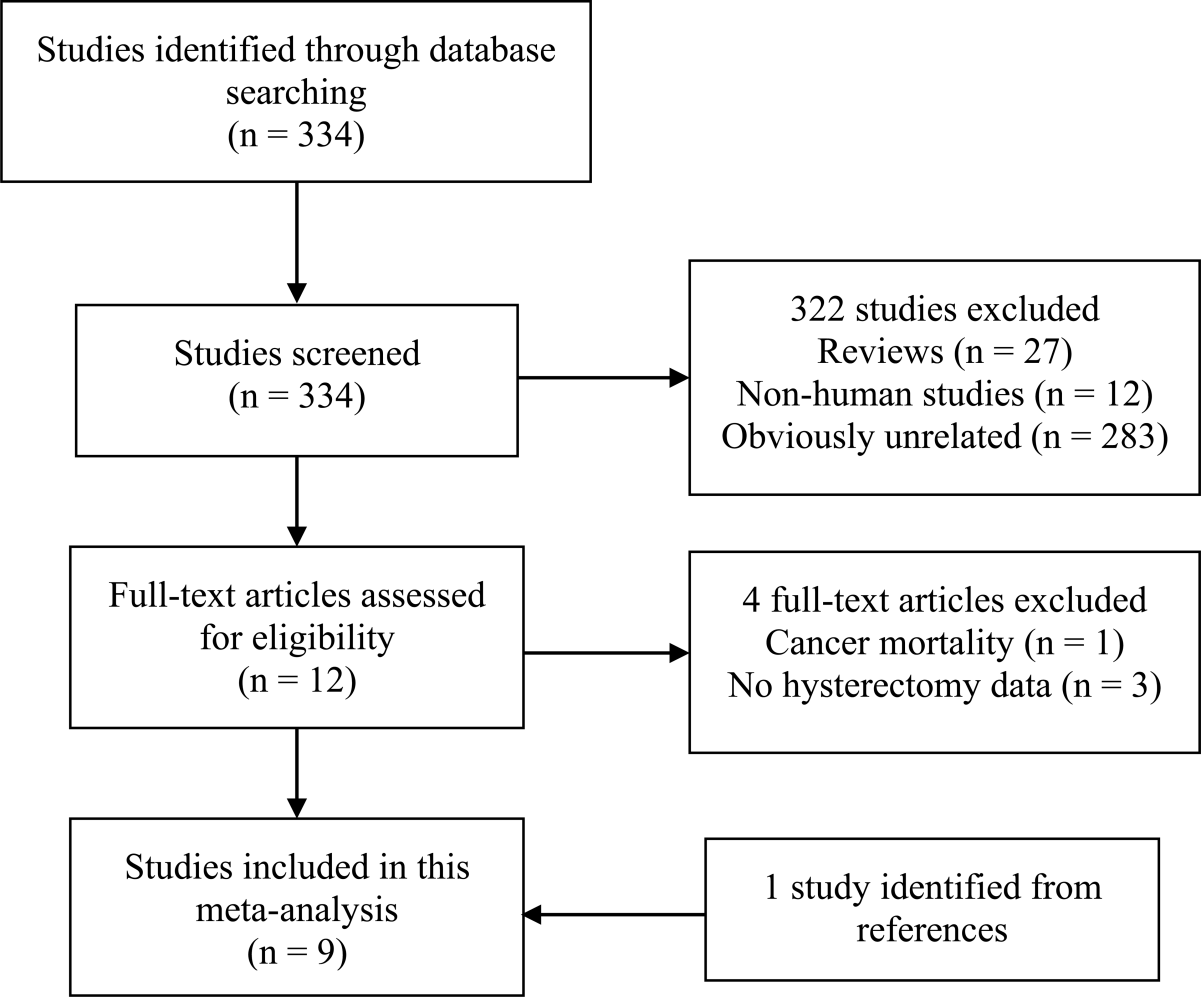
Literature search and selection process.

The detailed process of the literature search and study selection was presented in a flow diagram (**Figure 1**). Briefly, after removing duplications, the search strategy identified 334 articles. Of these, the majority were excluded after the first round of titles and abstract screening, mainly because they were reviews, non-human studies, or obviously not relevant to our analysis. After full-text review of 13 papers, 4 studies were excluded for the reasons as follows: cancer mortality as the outcome (23); the exposure not including hysterectomy (24–26). Thus, a total of 9 studies with 10 cohorts (9–15, 27, 28) were included in this meta-analysis. Of note, Karami et al’s study (14), based on NIH-AARP and PLCO cohorts, was regarded as two independent cohorts.

### Study characteristics

These cohorts were performed in the following regions: America (n = 6), Europe (n = 3), and Australia (n = 1). A total of approximately 240 million participants with 4752 cases were included in these studies. These cohort studies were published between 1997 and 2022, of which three large cohorts (15, 27, 28) were published in the recent two years, and were not included in a previous meta-analysis (29). Information on hysterectomy was obtained by self-administrated questionnaire or medical records. The outcome was confirmed by linking to cancer registry or medical records. Adjustments were made for potential confounding of one or more factors in all studies. The study quality, as assessed by the NOS, ranged from 6 to 8, with a mean value of 7.2 (**Supplemental Table S1**). The detailed information of the studies at baseline are presented in **Table 1**.

**Table 1:** Main characteristic of included studies.

First author	Year	Cohort name	Region	Study period	Age (y)	Participants	Cases	Follow-up (y)	Exposure	Outcome
Schouten et al.	2022	Netherlands Cohort Study	Netherlands	1986-2006	55-69	2280	204	20.3	Questionnaire	Cancer Registry
Wilson et al.	2021	Australian Data Linkage Study	Australia	1988-2014	Over 18	839332	1094	27	Medical records	Cancer Registry
Luo et al.	2021	Women’s Health Initiative	United States	1993-2010	50-79	144599	583	15.9	Questionnaire	Medical records
Karami-1 et al.	2013	NIH-AARP	United States	1995-2006	Median: 62.3	210300	601	11.2	Questionnaire	Cancer Registry
Karami-2 et al.	2013	PLCO	United States	1993-2010	Median: 63.1	73652	191	14.2	Questionnaire	Medical records
Altman et al.	2010	Swedish Health Care Registers	Sweden	1973-2003	Over 18	842233	1352	11.2/11.6	Medical records	Cancer Register
Lee et al.	2009	Nurses’ Health Study	United States	1976-2004	30-55	118219	247	28	Questionnaire	Medical records
Setiawan et al.	2009	Multiethnic Cohort	United States	1993-2005	45-75	106036	229	10.6	Questionnaire	Cancer Register
Molokwu et al.	2007	Iowa Women’s Health Study	United States	1986-2003	55-69	37440	165	15.7	Questionnaire	Cancer Register
Luoto et al.	1997	Finnish Mass Screening Registry	Finland	1967-1993	35-50	50734	86	20.5	Questionnaire	Cancer Register

y, year; PLCO, Prostate, Lung, Colorectal and Ovarian.

### Hysterectomy and risk of kidney cancer

**Figure 2:**
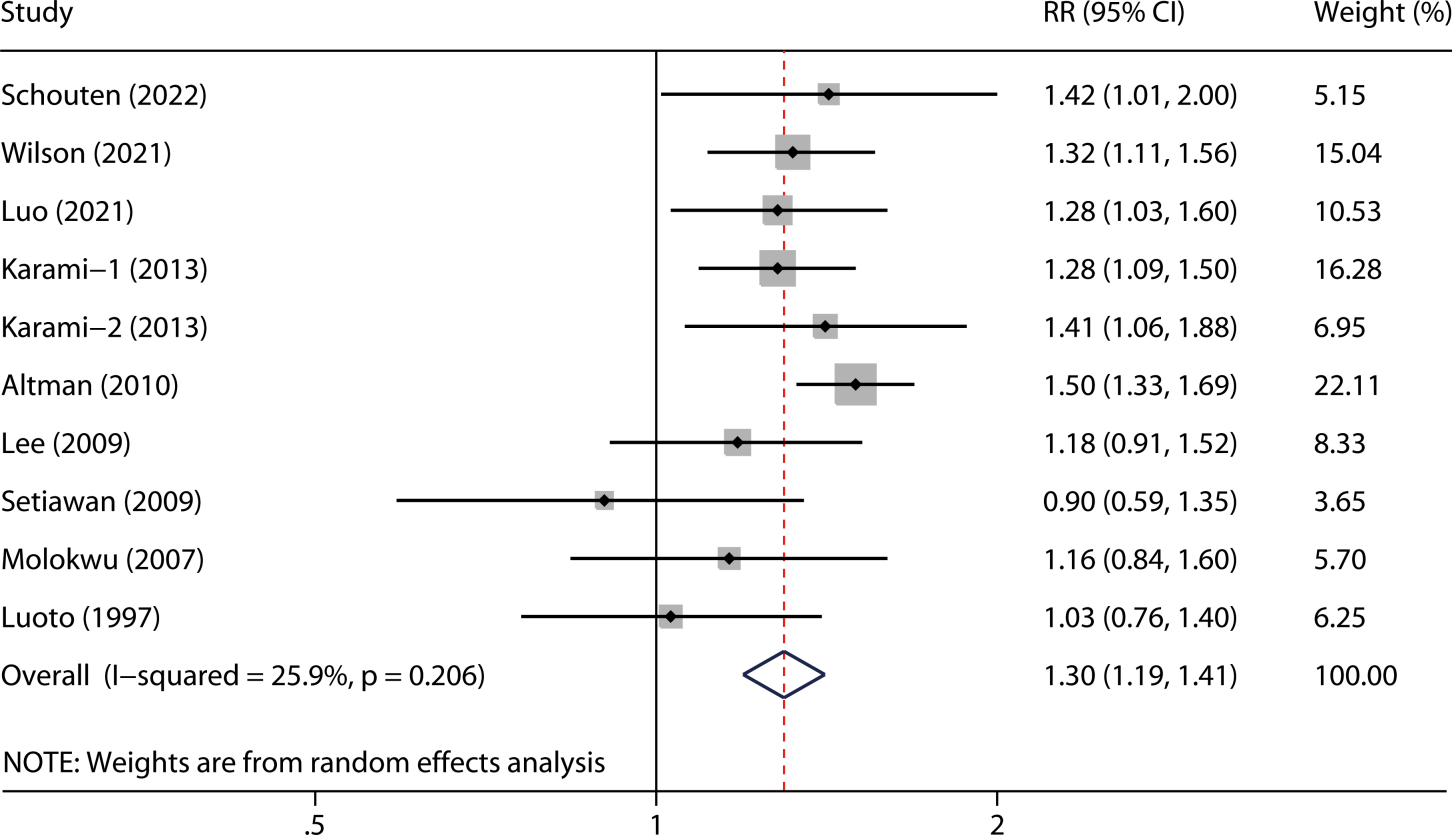
Summary of meta-analysis of hysterectomy and kidney cancer risk.

The overall RR with its 95% CI showed a statistically significant positive association between hysterectomy and kidney cancer risk (**Figure 2**, RR 1.30, 95% CI 1.19-1.41). No obvious heterogeneity was observed among the included studies (*P* = 0.206 for heterogeneity; I^2^ = 25.9%).

### Subgroup analysis

A significant association between hysterectomy and risk of kidney cancer was observed in most subgroups based on geographical region, publication year, duration of follow-up, number of participants and number of cases, except for studies published before 2010 (**Table 2**). No significant interactions were observed for these factors in all analyses based on meta-regression models (all *P* for interaction > 0.05).

**Table 2:** Subgroup analyses for the relationship between hysterectomy and kidney cancer.

Factors Stratified	No. of cohorts	RR (95% CI)	p for interaction	Q	*P*	I^2^, %
All studies	10	1.30 (1.19-1.41)		12.14	0.206	25.9
Location			0.510			
Europe	3	1.33 (1.06-1.67)		5.04	0.080	60.3
United States	6	1.24 (1.13-1.37)		3.61	0.606	0
Publication year			0.517			
> 2010	5	1.32 (1.20-1.44)		0.59	0.964	0
≤ 2010	5	1.19 (0.97-1.45)		11.51	0.021	65.3
Follow-up			0.264			
> 15 years	6	1.25 (1.13-1.38)		2.92	0.713	0
≤ 15 years	4	1.33 (1.14-1.56)		6.82	0.078	56.0
Participants, n			0.597			
> 100,000	6	1.31 (1.18-1.45)		8.41	0.135	40.6
≤ 100,000	4	1.24 (1.06-1.45)		2.96	0.398	0
Cases, n			0.122			
> 500	4	1.37 (1.26-1.49)		3.42	0.415	0.2
≤ 500	6	1.19 (1.05-1.35)		5.01	0.331	12.4

No., number; RR, relative risk; CI, confidence interval.

### Sensitivity analysis and publication bias

**Figure 3:**
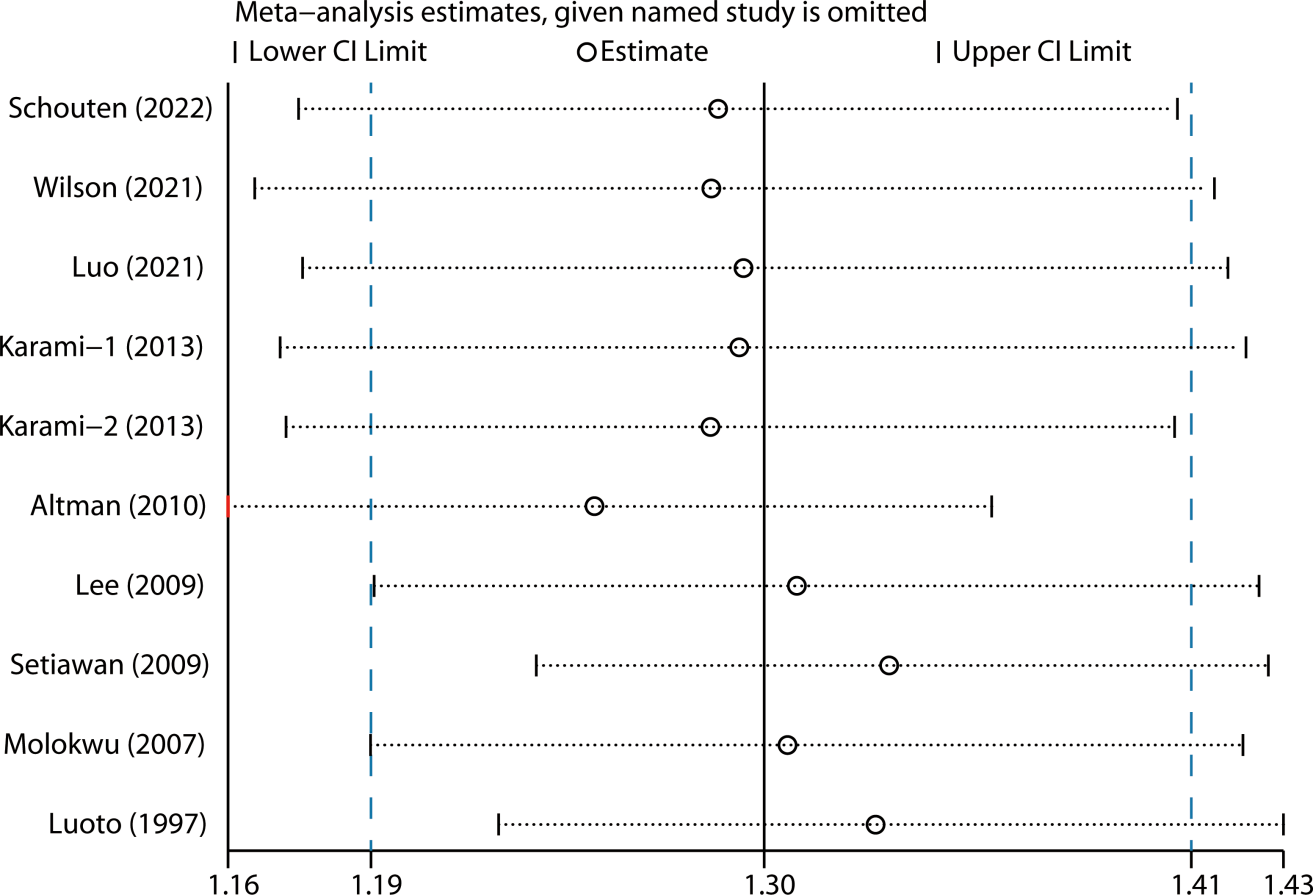
Sensitivity analysis by removing each study in turn and then repeating the pooled analysis.

**Figure 4:**
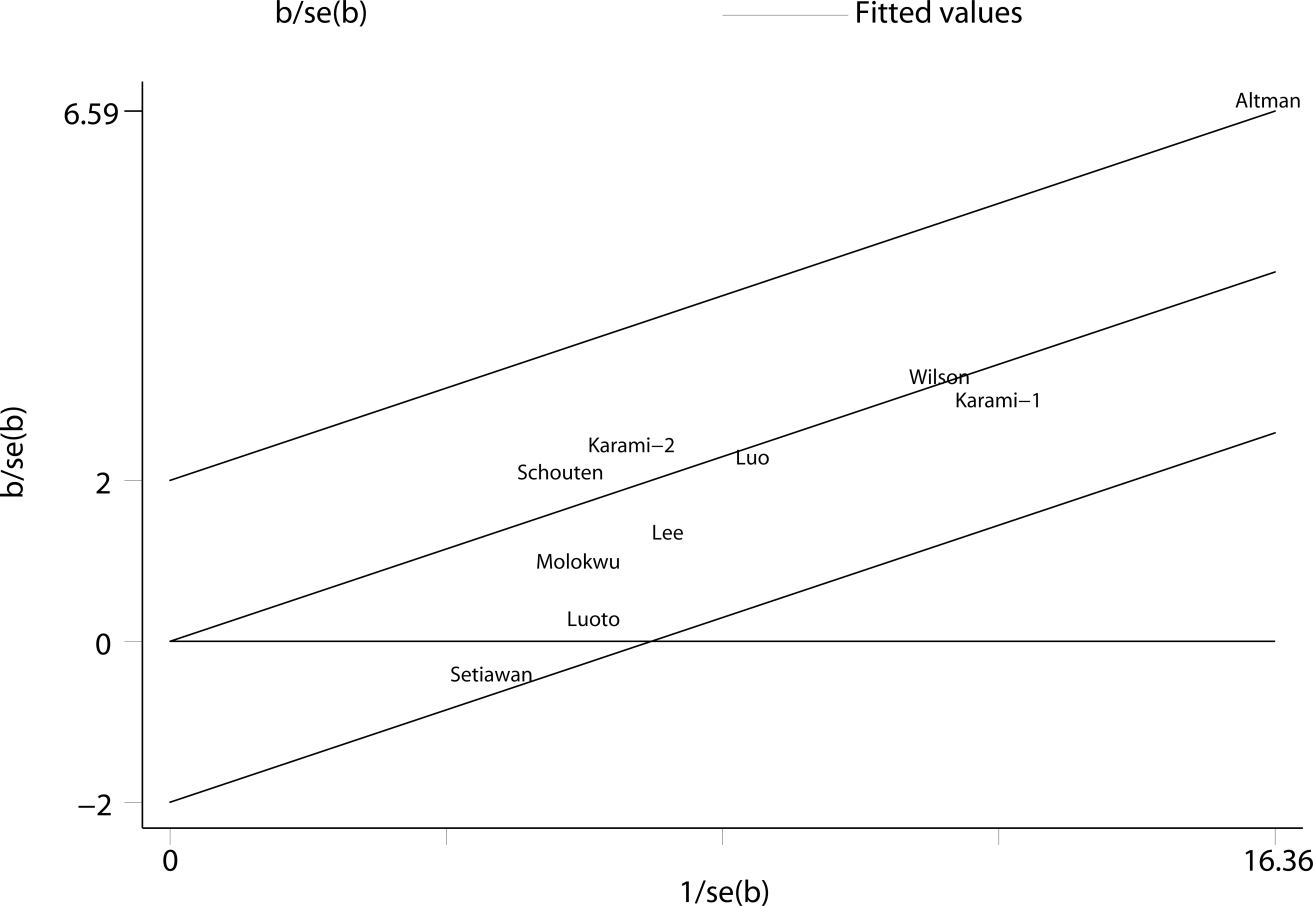
Galbraith plot analysis indicated that Altman et al.’s study was the major source of heterogeneity.

**Figure 5:**
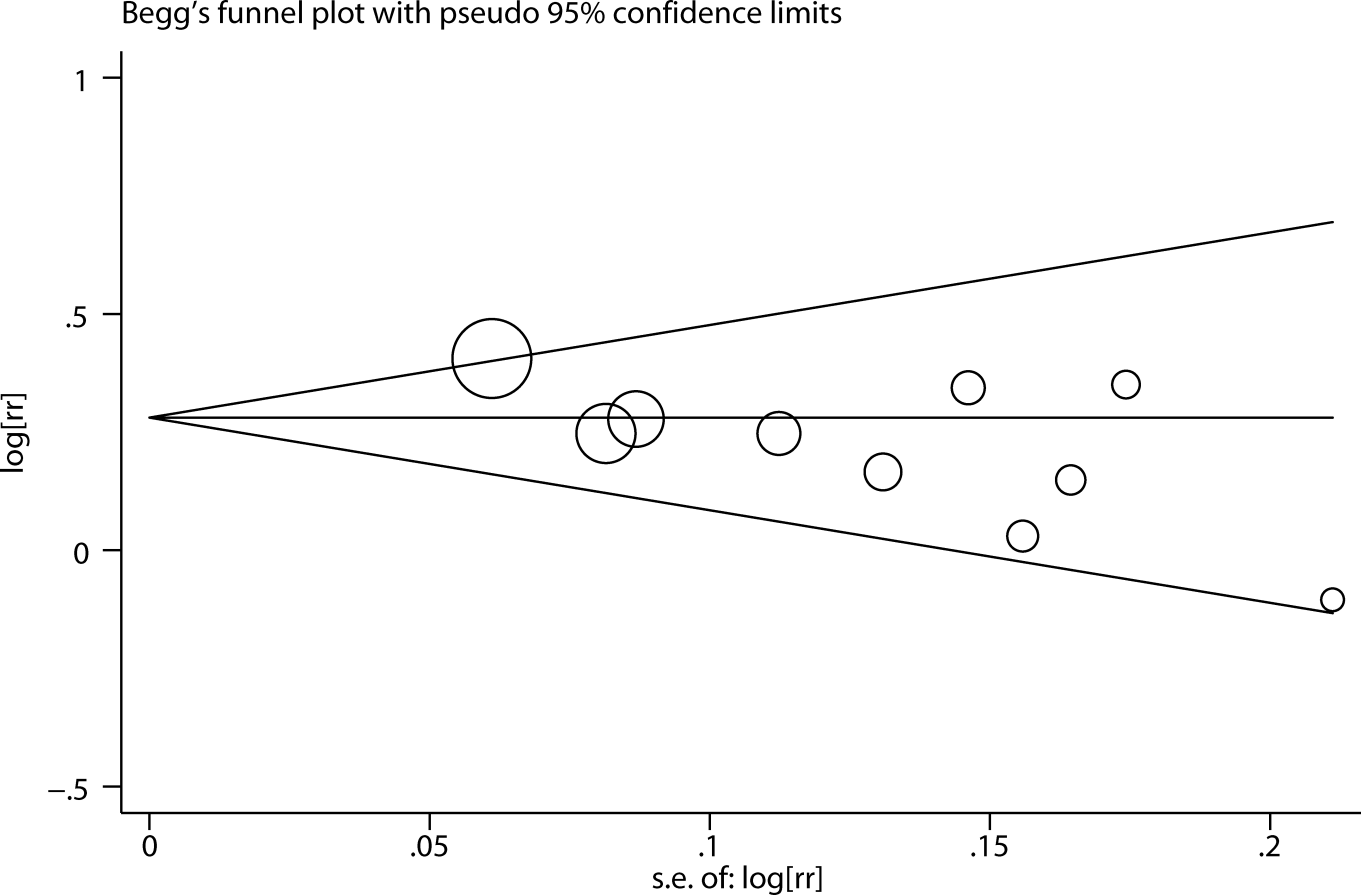
Publication bias analysis with a Begg’s test funnel.

We performed two types of sensitivity analyses. First, the impact of each study on the summary RR was evaluated by repeating the meta-analysis after omitting one study at a time. The exclusion of any single study did not substantially alter the pooled RR (**Figure 3**). Second, Galbraith plot analysis indicated that Altman et al.’s study was the major source of heterogeneity (**Figure 4**). After excluding this study, the heterogeneity was dramatically reduced, with I^2^ of from 25.9% (*P* = 0.206) to 0.0% (*P* = 0.636), while the association maintained significant (RR = 1.25, 95% CI 1.16-1.36). There was some degree of publication bias with Egger’s test (*P* = 0.017), whereas no evidence of publication bias was identified from Begg’s test (*P* = 0.152, **Figure 5**).

## Discussion

The findings from this meta-analysis of cohort studies indicated that hysterectomy was significantly associated with a 30% higher risk of kidney cancer, and the result was robust with no obvious heterogeneity observed across the included studies. Sensitivity analyses also showed similar results.

The results of our study were consistent with a previous meta-analysis published in 2014 (29), which also reported that hysterectomy was significantly associated with kidney cancer (RR 1.29, 95% CI 1.16-1.43). However, this study included both case-control and cohort studies. Case-control studies may be prone to select and recall bias. In addition, only seven cohort studiers were available in this meta-analysis. By contrast, our study was based on cohort studies and included the recent three large-scale cohort studies (11–13) with longer follow-up, which enhanced the statistical power.

Previous studies which reported a positive association between hysterectomy and kidney cancer risk have been criticized for the issue of detection bias. Detection bias may partially explain the positive results since women with a history of hysterectomy may seek medical care and exanimation more actively than those without, and thus had a higher chance to be detected with kidney cancer. However, detection bias may contribute to a higher short-term cancer incidence relative to the surgical procedure, but this may not still come into play decades after the surgery. In fact, this association did not change substantially in studies which performed subgroup analyses according to the time since hysterectomy or age at hysterectomy (13, 27).

It remains unclear what biological mechanisms would mediate the association between hysterectomy and kidney cancer risk. Hysterectomy may cause renal damage or pelvic anatomy changes. A high incidence of post-renal obstruction following hysterectomy has been reported in previous studies. Hydronephrosis could appear after hysterectomy even without any obvious intraoperative ureteral injury (14, 30, 31). It may result from the twisting and constricting of the distal ureter caused by pelvic anatomy changes after a hysterectomy. Ureteropelvic junction obstruction and persistent subclinical hydronephrosis after hysterectomy may be involved in renal cell proliferation and cancer transition of the renal parenchyma (32). Another possible explanation for the association between hysterectomy and risk of kidney cancer may attribute to estrogen and progesterone replacement therapy, which was commonly used among women who have undergone a hysterectomy. Abnormal endocrine stimulation played a significant role in kidney cancer pathophysiology (33). Progesterone has been reported to inhibit the kidneys’ ability to filter out toxins (34). Estrogen receptor α (ERα) overexpression increased the transcriptional factor activity of HIF-1α and inhibition of ER-α signaling in VHL-deficient cancer cells could suppress tumor development (33, 35). Recent studies also indicated that the estrogen receptor beta (ERβ) could affect the progression of renal cancer (36).

This study had several limitations. First, the confounding bias that was inherent in the included studies cannot be excluded. Inadequate control or adjustment for confounders may bias the results in either direction, leading to underestimation or overestimation of the risk estimates (37). Second, because of the various types of risk estimates and time intervals used by the original studies (**Supplemental Table S2**), we were unable to assess the impact of time since hysterectomy and age at hysterectomy on the association between hysterectomy and kidney cancer risk. Future prospective studies with detailed data on these could provide further insight into the relationship. Third, significant publication bias was observed with the Egger’s test. Although we performed a throughout literature search, grey papers and studies with a small sample size or null result are less likely to be published.

Nevertheless, this study had some strengths. First, a total of approximately 240 million participants were included in this meta-analysis, which provided sufficient statistical power to evaluate the impact, if any, of hysterectomy on kidney cancer risk. Second, only cohort studies were included in this meta-analysis, which, to some extent, avoided the selection and recall bias typically existing in case-control studies. Lastly, no significant heterogeneity was found across the included studies, which indicated our findings were relatively robust.

## Conclusions

In summary, findings from this meta-analysis of cohort studies indicated that hysterectomy was positively associated with the risk of kidney cancer. Future large prospective studies with long term follow-ups are warranted to verify these findings, and the potential underlying molecular mechanism that links hysterectomy and kidney cancer incidence needs further exploration.

## Data availability statement

The original contributions presented in the study are included in the article/**Supplementary Material**. Further inquiries can be directed to the corresponding author.

## Author contributions

LY: project development, data collection and analysis, manuscript writing and editing. PY: data collection and analysis, manuscript writing. YL: project development, manuscript editing, and project supervising.
