# Supplementary material for: Is hysterectomy associated with kidney cancer risk? A meta-analysis of cohort studies
Source: Front Oncol. 2023 Jul 20;13:1181112. doi: 10.3389/fonc.2023.1181112 (PMC10397505; doi:10.3389/fonc.2023.1181112)
Supplement: Supplementary file 1 [file Table_1.docx]

**Table S1**. Methodological quality of studies evaluated by the Newcastle-Ottawa Scale (NOS)

| Included Studies^1^ | Representativeness of the exposed  cohort | Selection of the  unexposed  cohort | Exposure  assessment | Outcome not present at baseline | Comparability ^2^ | Outcome  assessment | Follow-up long enough ^3^ | Adequacy  of follow-up ^4^ | Summary  scores |
| --- | --- | --- | --- | --- | --- | --- | --- | --- | --- |
| Schouten et al., 2022 | ☆ | ☆ |  | ☆ | ☆☆ | ☆ | ☆ | ☆ | 8 |
| Wilson et al., 2021 | ☆ | ☆ | ☆ | ☆ | ☆ | ☆ | ☆ | ☆ | 8 |
| Luo et al., 2021 | ☆ | ☆ |  | ☆ | ☆☆ | ☆ | ☆ | ☆ | 8 |
| Karami-1., 2013 | ☆ | ☆ |  | ☆ | ☆☆ | ☆ |  | ☆ | 7 |
| Karami-2, 2013 | ☆ | ☆ |  | ☆ | ☆☆ | ☆ |  | ☆ | 7 |
| Altman et al., 2010 | ☆ | ☆ | ☆ | ☆ |  | ☆ |  | ☆ | 6 |
| Lee et al., 2009 | ☆ | ☆ |  | ☆ | ☆☆ | ☆ | ☆ | ☆ | 8 |
| Setiawan et al., 2009 | ☆ | ☆ |  | ☆ | ☆☆ | ☆ |  | ☆ | 8 |
| Molokwu et al., 2007 | ☆ | ☆ |  | ☆ | ☆ | ☆ | ☆ | ☆ | 7 |
| Luoto et al., 1997 | ☆ | ☆ |  | ☆ |  | ☆ | ☆ | ☆ | 6 |

^1^ A study can be awarded a maximum of one star for each item except the item ‘Comparability’.

^2^ A maximum of two stars can be awarded for this item. Studies adjusting for smoking status and body size received one star while studies additionally adjusting for other important confounders received an additional star.

^3^ A cohort study with a follow-up time more than 15 years was assigned one star.

^4^ A cohort study with a follow-up rate more than 75% was assigned one star.
