# Supplementary material for: Is hysterectomy associated with kidney cancer risk? A meta-analysis of cohort studies
Source: Front Oncol. 2023 Jul 20;13:1181112. doi: 10.3389/fonc.2023.1181112 (PMC10397505; doi:10.3389/fonc.2023.1181112)
Supplement: Supplementary file 2 [file Table_2.docx]

**Supplemental Table S2 Studies that assessed the impact of time since hysterectomy and age at**

**hysterectomy**

| Altman et al. | Age at Hysterectomy | < 45 | 45-49 | 50-57 | ≥ 58 |
| --- | --- | --- | --- | --- | --- |
|  | RR (95% CI) | 2.03 (1.55-2.67) | 1.47 (1.17-1.85) | 1.29 (1.01-1.64) | 1.43 (1.12-1.83) |
| Karami et al.-1 | Age at Hysterectomy | < 40 | 40–44 | 45–49 | ≥50 |
|  | R (95% CI) | 1.00 | 0.90 (0.66-1.22) | 1.12 (0.83-1.52) | 1.06 (0.72-1.56) |
| Karami et al.-2 | Age at Hysterectomy | < 40 | 40–44 | 45–49 | ≥50 |
|  | R (95% CI) | 1.00 | 0.74 (0.41-1.32) | 1.21 (0.71-2.07) | 0.77 (0.41- 1.44) |
| Luo et al. | Age at Hysterectomy | < 40 | 40–49 | 50–54 | ≥55 |
|  | R (95% CI) | 1.35 (1.01–1.80) | 1.19 (0.91-1.57) | 1.06 (0.70–1.60) | 1.41 (1.04–1.91) |
| Wilson et al. | Age at Hysterectomy | < 45 | 45–54 | ≥55 |  |
|  | R (95% CI) | 1.47 (1.21–1.78) | 1.08 (0.82-1.42) | 1.07 (0.67–1.63) |  |
| Altman et al. | Time Since Hysterectomy | 0-10 | 11-20 | >20 |  |
|  | R (95% CI) | 1.50 (1.26-1.78) | 1.49 (1.22-1.82) | 1.51 (1.05-2.16) |  |
